# A Clinician's Dictionary of Pathogenic Microorganisms

**DOI:** 10.3201/eid1009.040540

**Published:** 2004-09

**Authors:** Didier Raoult

**Affiliations:** * Unité des Rickettsies, Marseille, France

**Keywords:** A Clinician's Dictionary of Pathogenic Microorganisms, James H. Jorgensen, Michael A. Pfeller

This dictionary of pathogenic microorganisms, published by the American Society for Microbiology, is simple and useful. This book is divided in four sections, bacteria, fungi, parasites, and viruses. Each organism is presented alphabetically in its section. Older names are mentioned and connected with current names. A brief bibliography is also provided at the end of each chapter.

The emergence of new infectious agents in the last 2 decades makes it difficult for clinicians to recognize new diseases and new names. A memorandum to address this matter have been useful. Moreover, the genomic revolution has caused a taxonomic revolution; this is specifically true for bacteriology. For example, 16S rRNA sequencing allowed reclassification of many pathogenic organisms and descriptions of many others. These advances is genomic knowledge have brought about many changes in the names of pathogenic microorganisms, evidenced here by the authors' devoting the largest part of the book to bacteria.

The information provided, although very brief, is usually complete enough to provide a basic understanding of the microorganism. Many new organisms such as *Ehrlichia* and monkeypox viruses, as well as emerging diseases such as severe acute respiratory syndrome, are included.

This book provides basic information clinicians need for a quick reference book. It largely succeeds in this attempt and may be very useful as a pocket book for nonspecialists at the patient's bedside. I recommend it for general practitioners and health professionals.

